# “EXPECT THE UNEXPECTED”: CUSTODIAL GRANDPARENTS’ PERSPECTIVES ON RAISING GRANDCHILDREN DURING COVID-19

**DOI:** 10.1093/geroni/igad104.0858

**Published:** 2023-12-21

**Authors:** Christine Fruhauf, Deborah Whitley, Susan Kelley, Andrea Smith, Youjung Lee, Heidi Tseng, Bert Hayslip

**Affiliations:** Colorado State University, Fort Collins, Colorado, United States; Georgia State University, Atlanta, Georgia, United States; Georgia State University, Atlanta, Georgia, United States; Western Michigan University, Ada, Michigan, United States; Binghamton University, State University of New York, Binghamton, New York, United States; Colorado State University, Fort Collins, Colorado, United States; University of North Texas, Denton, Texas, United States

## Abstract

Managing COVID-19 demands may exacerbate caregiving responsibilities of custodial grandparents raising grandchildren. This presentation aims to discuss qualitative data obtained from 14 custodial grandmothers who participated in virtual focus groups to understand their parenting experiences in conjunction with meeting COVID-19 demands. Participants were recruited from a larger sample (N=145, age range 30-79 years, Mage = 61.4 years, SD = 8.5) of custodial grandparents who completed a quantitative survey on COVID-19 stressors. Guided by McCubbins and Patterson’s Family Stress and Adaptation model, three researchers conducted open coding of the focus group data. The analysis revealed distinguishing patterns related to managing challenges of grandchild caregiving brought on by COVID-19. Some grandparents discussed the demands of COVID-19 as difficult, but not as challenging as the day-to-day experiences of raising grandchildren. This was reflected when grandparents discussed raising grandchildren with special needs in the pandemic environment, advocating with schools to adjust virtual learning expectations, managing custody cases and social service home visits while ‘protecting’ grandchildren and themselves from COVID-19 exposure. Grandparents stated they did not change their parenting style to manage COVID-19 demands, yet they discovered a stronger, internal commitment to raising grandchildren. Grandparents reflected when isolating they enjoyed engaging in different and new activities with their grandchildren while becoming closer to them as a result. Findings suggest that challenges of raising grandchildren were heightened in the context of COVID-19 demands. Although grandparents experienced losses during the pandemic, they also reflected on gains related to their resourcefulness and resiliency in managing stressors.

